# Standardizing T2 measurements for the quantitative assessment of regional myocardial edema

**DOI:** 10.1186/1532-429X-13-S1-P121

**Published:** 2011-02-02

**Authors:** Iacopo Carbone, Helene Childs, Yoko Mikami, Vanessa M Ferreira, Ingo Eitel, Matthias G Friedrich

**Affiliations:** 1 University of Calgary, Calgary, AB, Canada; 2 Heart Center - University Leipzig, Leipzig, Germany

## Introduction

Accurate analysis of T2-weighted magnetic resonance images (MRI) is crucial for myocardial edema (ME) assessment. In view of frequent artifacts from motion, slow flow and field inhomogenities affecting infarcted and remote myocardium (ReM), several approaches can be used to evaluate ME: 1) visual assessment, 2) signal intensity (SI) > 2 SDs of ReM or 3) normalizing myocardial SI to that of a skeletal muscle: T2 Ratio. Currently, there is no standardized approach to a (semi-) quantitative edema assessment using T2-weighted imaging.

## Purpose

The purpose of this study is to compare different approaches for the analysis of T2-weighted images in patients with late reperfused myocardial infarction (MI) for reproducibility and agreement with the extent of MI, using LGE as a standard of truth.

## Methods

Thirty patients with acute late reperfused MI (time to reperfusion > 200 minutes) who underwent cardiac MRI at 1.5 T within 5 days from onset of symptoms were retrospectively studied. The MRI protocol included b-SSFP cine, T2-STIR imaging using a body coil and Late Gadolinium Enhancement (LGE) imaging. Ten patients demonstrated microvascular obstruction (MVO) on LGE images and were analyzed separately. Infarct size was determined on LGE images using a threshold of 2SD and 5SD above the SI of ReM. Regions of interest were traced manually; edema size was measured using thresholds of 2, 3 and 5SDs above the SI of ReM, as well as using the T2 Ratio.

## Results

When using LGE with a cutoff of 2SD, the LGE area with high signal intensity was larger than the area with high T2 signal intensity (2SD). There was a very good agreement between LGE/5SD and T2 ratio in both, patients without MVO (r=0.826, p < 0.001; no significant difference, p=0.180) and with MVO (r=0.996, p < 0.001; no significant difference, p=0.224). Infarct size defined by LGE/5SD was significantly smaller than edema size using thresholds of 2SD and significantly larger using a 5SD threshold (both p<0.001).

**Figure 1:**
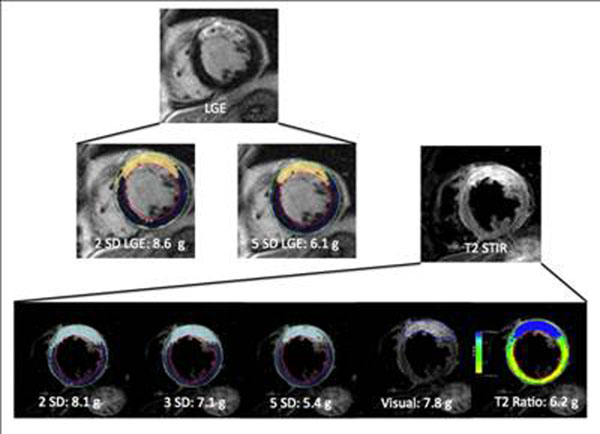


In contrast, there was no correlation between LGE/2SD and T2 measurements. Figure 1.

## Conclusion

Using LGE/5SD in patients with late reperfusion acute myocardial infarction, a normalized T2 signal intensity ratio appears to be the most accurate approach for the evaluation of the size of infarct-related edema.

